# Evaluation Metrics for Augmented Reality in Neurosurgical Preoperative Planning, Surgical Navigation, and Surgical Treatment Guidance: A Systematic Review

**DOI:** 10.1227/ons.0000000000001009

**Published:** 2023-12-26

**Authors:** Tessa M. Kos, Elisa Colombo, L. Wilbert Bartels, Pierre A. Robe, Tristan P. C. van Doormaal

**Affiliations:** * Image Sciences Institute, University Medical Center Utrecht, Utrecht, The Netherlands;; ‡ Department of Neurosurgery, Clinical Neuroscience Center, Universitätsspital Zürich, Zurich, The Netherlands;; § Department of Neurosurgery, University Medical Center Utrecht, Utrecht, The Netherlands

**Keywords:** Augmented reality, Mixed reality, Neurosurgery, 3D visualization, Evaluation metrics

## Abstract

**BACKGROUND AND OBJECTIVE:**

Recent years have shown an advancement in the development of augmented reality (AR) technologies for preoperative visualization, surgical navigation, and intraoperative guidance for neurosurgery. However, proving added value for AR in clinical practice is challenging, partly because of a lack of standardized evaluation metrics. We performed a systematic review to provide an overview of the reported evaluation metrics for AR technologies in neurosurgical practice and to establish a foundation for assessment and comparison of such technologies.

**METHODS:**

PubMed, Embase, and Cochrane were searched systematically for publications on assessment of AR for cranial neurosurgery on September 22, 2022. The findings were reported according to the Preferred Reporting Items for Systematic Reviews and Meta-Analyses guidelines.

**RESULTS:**

The systematic search yielded 830 publications; 114 were screened full text, and 80 were included for analysis. Among the included studies, 5% dealt with preoperative visualization using AR, with user perception as the most frequently reported metric. The majority (75%) researched AR technology for surgical navigation, with registration accuracy, clinical outcome, and time measurements as the most frequently reported metrics. In addition, 20% studied the use of AR for intraoperative guidance, with registration accuracy, task outcome, and user perception as the most frequently reported metrics.

**CONCLUSION:**

For quality benchmarking of AR technologies in neurosurgery, evaluation metrics should be specific to the risk profile and clinical objectives of the technology. A key focus should be on using validated questionnaires to assess user perception; ensuring clear and unambiguous reporting of registration accuracy, precision, robustness, and system stability; and accurately measuring task performance in clinical studies. We provided an overview suggesting which evaluation metrics to use per AR application and innovation phase, aiming to improve the assessment of added value of AR for neurosurgical practice and to facilitate the integration in the clinical workflow.

**ABBREVIATIONS:**
AR: augmented reality
FDA: Food and Drug Administration
FRE: fiducial registration error
MxR: mixed reality
NASA-TLX: National Aeronautics and Space Administration Task Load Index
OR: operating room
TRE: target registration error.

Augmented reality (AR) is a technology that enables the integration of virtual and real environments, falling within the broader category of mixed reality (MxR) technologies. MxR encompasses a range of technologies on the reality–virtuality continuum, offering varying degrees of consolidation between the real and virtual world (Figure 1).^1^ AR technology finds numerous medical applications, including 3-dimensional (3D) visualization and interaction with medical imaging data. Within the domain of neurosurgery, AR technology can provide valuable 3D insights into relevant anatomic structures and their relationships. Such insights can aid in surgical planning and guidance and can also be used for surgical training, medical education, and patient information purposes.^2^

**FIGURE 1.**
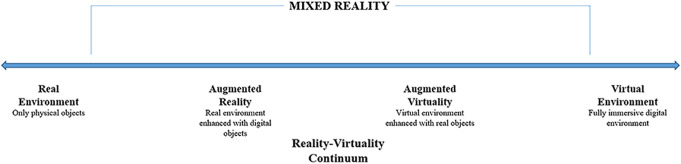
The reality–virtuality continuum, as described by Milgram et al.^1^

Several review articles have discussed the use of AR in neurosurgery, covering various key areas including education, spine surgery, surgical navigation, vascular and neuro-oncological surgery, and surgical planning.^2,3^ They have also described the different indications and AR applications in neurosurgical practice.^4,5^ Despite a considerable growth in the number of publications on AR applications for neurosurgery over the past few years and rapid technical development of these technologies, the integration of these technologies in clinical practice remains lagging. Regulatory approval, clinical acceptance, and certification of these devices for implementation in the operating room (OR) require rigorous evaluation of technical safety and effectiveness. The lack of standardized evaluation methods complicates quality assessment, risk analysis, and comparison of AR for different neurosurgical applications. Recognizing this issue, the US Food and Drug Administration (FDA) has initiated efforts to establish standardized evaluation methodologies. They have formed an FDA working group for implementation of MxR technologies in the OR^6^ and organized a public workshop specifically addressing the challenges associated with evaluating MxR technologies.^2,3,7^ These efforts are essential for ensuring the safe and effective adoption of MxR technologies in clinical practice.

Therefore, the objectives of this review are two-fold: (1) to provide an overview of the currently used evaluation metrics for AR tools for the purpose of preoperative neurosurgical planning, intraoperative guidance, and surgical navigation and (2) to provide a basis for a consistent evaluation framework for technical and clinical use assessment and valorization of these techniques.

## METHODS

### Search Strategy and Selection Process

A systematic review of the outcome metrics used for the assessment of AR techniques for preoperative planning and intraoperative guidance in neurosurgery was performed in accordance with the Preferred Reporting Items for Systematic reviews and Meta-Analyses guidelines.^8^ A systematic search was conducted in the databases PubMed, Embase, and Cochrane on September 22, 2022. The respective search strings are presented in **Supplemental Digital Content 1**, http://links.lww.com/ONS/B47, Search String. This review was not registered in any systematic review database. Publications found in this search were checked for duplicates and assessed for eligibility by two independent authors. Inclusion criteria were as follows: (1) publications describing the application or evaluation of an AR for preoperative planning and/or intraoperative use for human cranial neurosurgical procedures, (2) publications with full English text, (3) publications describing original research by the authors, and (4) publications that have been peer-reviewed. Exclusion criteria were as follows: (1) publications in which AR for neurosurgery was not the main research subject or no evaluation methods or quality metrics were mentioned, (2) publications describing AR applications for surgical training or education, and (3) publications describing non-AR applications.

### Data Extraction

Each included publication was reviewed by the first author. Information on the authors, year of publication, and surgical purpose was extracted. For our further data extraction, we have adapted the taxonomy presented by Gsaxner et al^9^ specifically for AR applications with neurosurgical purposes to categorize each publication according to (1) AR application, (2) type of AR device, and (3) evaluation metrics.

#### AR Device

The following four categories were used to categorize the AR devices: (1) head-mounted devices showing a hologram, (2) tablets and smartphones showing a 3D scene, (3) micro- or endoscopic field-of-view image overlays showing a projection through the microscope or on the external screen, and (4) external projection system/Laser Imaging Detection And Ranging camera, which are capable of measuring the varying depths of their environment.

#### Evaluation Metrics

Five categories of evaluation metrics were identified and used: (1) registration accuracy, (2) clinical outcome, (3) time measurements, (4) technical reliability, and (5) user perception.

#### Applications of AR for Neurosurgical Procedures

Three application categories with increasing risk profiles were used: (1) AR for preoperative visualization, where the AR application serves as a method for data visualization; (2) AR surgical navigation, where registration of the virtual on the real environment is required; and (3) AR for intraoperative guidance, where next to image guidance, tracking of surgical tools is also required.

## RESULTS

### Search Results

**FIGURE 2.**
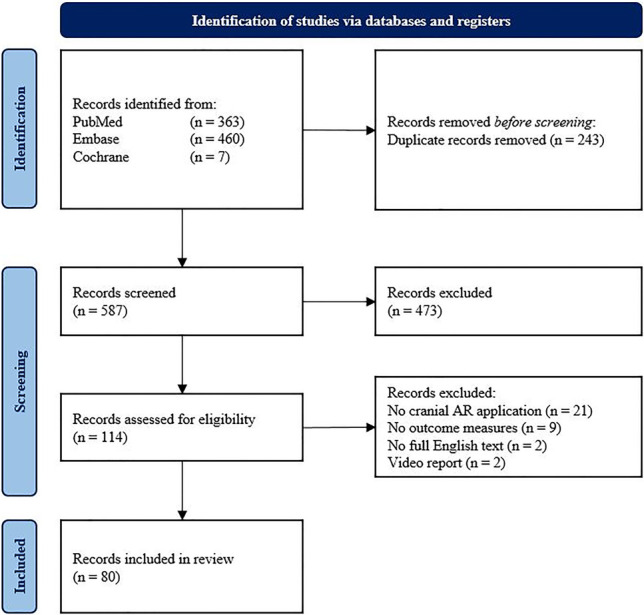
Flowchart summarizing the study selection process according to the Preferred Reporting Items for Systematic reviews and Meta-Analyses guidelines.^8^

The systematic search yielded 830 publications. After duplicate removal, 587 records remained for screening of title and abstract. Articles were mainly excluded when they described an immersive virtual reality application instead of an AR application, for describing surgical training instead of intraoperative applications, or if there was no cranial application. One hundred and fourteen records were included for full-text eligibility assessment. After independent revision by two authors (TK, EC), 80 publications were included in this systematic review. Nine publications were excluded specifically for a lack of outcome measures (Figure 2).

### Study Demographics

**FIGURE 3.**
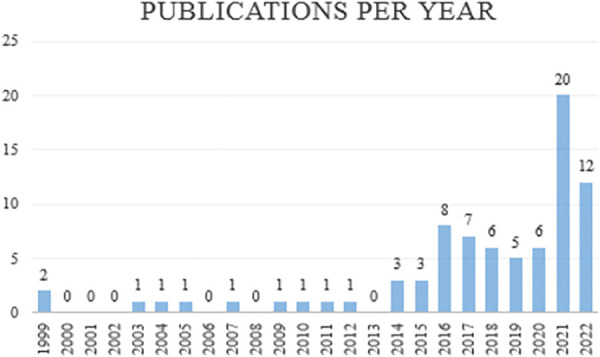
Publications per year, until search date 22 September 2022.

Figure 3 shows the number of included publications per year. It shows an overall increase in publications over time. The number in 2022 was reduced, possibly in part due to this review's search date.

### AR Device

**FIGURE 4.**
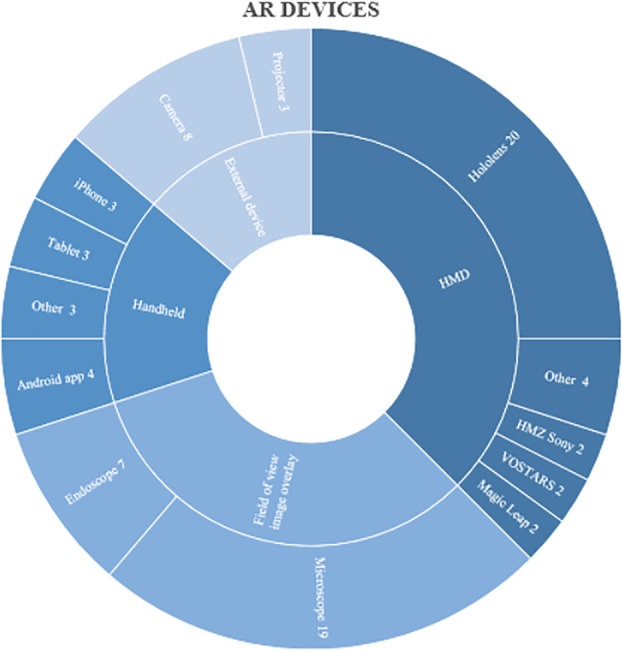
Distribution of AR devices used in the included studies. AR, augmented reality.

In 30 studies (38%), the use of a head-mounted device for neurosurgery was evaluated (**Supplemental Digital Content 2, Table 1**, http://links.lww.com/ONS/B48).^10-86^ In most of these studies, the HoloLens (Microsoft) was used for AR visualization. In 26 studies (32%), an image overlay, for either the microscopic or endoscopic field of view, was used. In 13 studies (16%) a tablet or smartphone was used for AR visualization. In 11 studies (14%) an external Laser Imaging Detection And Ranging camera or a projection device facilitated an AR visualization of the medical images. Figure 4 further specifies the distribution of the types of AR devices.

### Evaluation Metrics

Most of the studies reported on multiple evaluation metrics (**Supplemental Digital Content 3, Table 2**, http://links.lww.com/ONS/B49).^10-83,85-90^ Based on the results of this review, the following evaluation categories have been defined: (1) registration accuracy, (2) clinical outcome, (3) time measurements, (4) task performance, (5) technical reliability, and (6) user perception (Table).

**TABLE.** Evaluation Metric Frequency

Evaluation metric	Frequency			
	Preoperative planning	Surgical navigation	Intraoperative guidance	Total
Registration accuracy (63)				
Distance measure		19	3	22
Target registration error		12	2	14
Fiducial registration error		9	3	12
Visual assessment registration accuracy		9	1	10
Comparison of neuronavigation		3		3
Overlap measure		2		2
Time measurements (33)				
Registration time		11	2	13
Operative time		9		9
Planning time		5	1	6
Time for task		3	2	5
Technical reliability (9)				
Technical failures		5		5
Precision		2		2
Robustness		1		1
System latency	1			1
Task performance (29)				
Target point deviation		8	4	12
Task success rate		5	4	9
Target angle deviation		5		5
Task accuracy		2		2
Qualitative task performance evaluation			1	1
Clinical outcome (38)				
Complication occurrence	1	10	2	13
Tumor removal		8		8
General clinical outcome		4	1	5
Craniotomy size		2	1	3
Volume measurements		3		3
AR indication		2		2
Corresponding intraoperative findings			1	1
Recognition of unexpected findings			1	1
Surgical approach		1		1
Preservation structures		1		1
User perception (48)				
Usability	1	6	4	11
Usefulness		6	3	9
Performance evaluation	1	3	2	6
Ergonomics	1	4	1	6
Clinical feasibility	1	4		5
Task load (NASA-TLX)		2	1	3
Spatial aptitude		1	2	3
Depth perception			3	3
Trust in system		2		2

AR, augmented reality; NASA-TLX, National Aeronautics and Space Administration Task Load Index.

#### Registration Accuracy

The most frequently used evaluation metric for assessing the registration accuracy was the distance between the target object and the virtual object, reported in mm or in number of pixels, for which pixel size was provided in most studies. Different methods were used for measuring the distance between two points. A distance measure without further specification of the calculation method was provided in 22 studies. The target registration error (TRE) was described in 14 studies. The fiducial registration error (FRE) was described in 12 studies. In 10 studies, a visual assessment of the registration accuracy was described. Furthermore, in three studies, the accuracy of their AR system compared with the conventional neuronavigation system was described, and in two studies, an overlap measure of the structures of interest was described.

#### Time Measurements

The most frequently used time measurement was the registration time, which is defined as the time needed for registration of the AR model onto the real environment. The registration time was reported in 13 studies. In nine studies, the operative time when using an AR system was reported, in six studies, the preoperative planning time when using the AR system was reported, and in five studies, the time taken for performing a task while using the AR system was reported.

#### Technical Reliability

The technical reliability of the AR systems was evaluated in nine studies. In five studies, (a lack of) technical failures during AR use was reported. In two studies, the registration precision was reported, and in one study, the registration robustness of the AR system was reported, defined as the successful registration ratio. In one study, the system latency of the AR technology was reported.

#### Task Performance

Task performance metrics were reported for two tasks: placement of craniotomy burr holes and ventricular drains. The most frequently reported performance metric was the deviation from the target point, which was reported in 12 studies. The deviation from the target angle was reported in five studies. Task success rate was reported in nine studies, and a distance measure of craniotomy tracing accuracy was reported in two studies. In one study, a qualitative task-specific performance metric was reported, in this case for correct drain placement.

#### Clinical Outcome

In 13 studies, the occurrence of surgical complications was mentioned. In eight studies, tumor removal rates were reported. AR technologies were also assessed by healthy structure preservation, craniotomy size, change of surgical approach, recognition of unexpected findings, and general clinical outcome. In one study, the correspondence of AR with intraoperative findings was assessed, and in two studies, the indications for use of AR technology were mentioned. In three studies, tumor volume measurements were provided.

#### User Perception

Qualitative assessment of the use of AR technology was performed through various questionnaires. In 11 studies, a usability evaluation was conducted although the specific type of questionnaire or assessment varied. Although a differentiation was made between using a questionnaire or comments, in none of the studies, the used questions were specified. In nine studies, the usefulness of the AR system was assessed through a questionnaire or general user comments. The ergonomics of the system were evaluated separately in six studies, and the surgeons' spatial aptitude and National Aeronautics and Space Administration Task Load Index,^91^ scores were reported in three studies. Furthermore, in two studies, the surgeons' pre-task trust and post-task trust in the AR system were evaluated.

### Applications of AR for Neurosurgical Procedures

In four studies (5%), an AR application for preoperative visualization of imaging data was described (**Supplemental Digital Content 4, Table 3**, http://links.lww.com/ONS/B50).^10-83,85-90^ Their most frequently described evaluation metrics were user perception metrics (Table). Clinical outcome was mentioned in one study. The evaluation of an AR application for surgical navigation was described in 60 studies (75%). The most frequently described evaluation metrics were for registration accuracy, clinical outcome, and time measurements. An AR application for IGIs was described in 16 studies (20%). Their most frequently described evaluation metrics were for registration accuracy, task performance, and user perception.

## DISCUSSION

In this systematic review, 80 original research articles were assessed, with a focus on the evaluation metrics used for the AR technology. The results from this review revealed a large variability and low consistency in evaluation metrics used. As in other fields, like machine learning in medical image analysis, clinical benchmarking could facilitate comparison of different techniques and establish quality standards for the use of AR in clinical practice.

**FIGURE 5.**
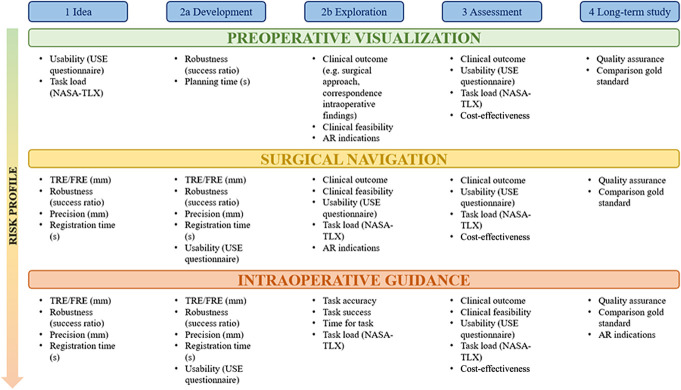
Overview of evaluation metrics per application and IDEAL^94^ innovation phase. AR, augmented reality; FRE, fiducial registration error; IDEAL, Idea, Development, Exploration, Assessment, Long-term study; NASA-TLX, National Aeronautics and Space Administration Task Load Index; TRE, target registration error USE, Usefulness, Satisfaction and Ease of Use.

Clinical benchmarking involves defining a specific clinical problem, using accompanying data sets, establishing an appropriate infrastructure, and evaluating the technology based on its ability to address the clinical problem.^92,93^ In addition, in the development of AR technology for neurosurgical practice, the Idea, Development, Exploration, Assessment, Long-term study (IDEAL) framework for surgical innovation^94^ could be taken into consideration, which provides a checklist structure suggesting evaluation study design and metrics. Similar to the development of surgical tools, the evaluation metrics used for assessing AR technologies depend on their use case and innovation phase. The three application categories defined in this review have increasing risk profiles in their application, requiring different types of assessment. An overview of the proposed evaluation metrics per application and innovation phase is presented in Figure 5. The evaluation of AR technologies used for preoperative visualization has primarily involved the assessment of user perception of the technology, which is essential for its implementation. User perception can be measured through various aspects, preferably using validated questionnaires. This review found that the system usability and usefulness were most frequently assessed although the evaluation methods were inconsistent and no validated questionnaires were used. To evaluate the usability of AR systems in clinical practice, we propose the use of the validated Usefulness, Satisfaction and Ease of Use (USE) questionnaire,^95^ which consists of 30 questions rated on a seven-point Likert scale, divided over the categories “Usefulness,” “Ease of Use,” “Ease of Learning,” and “Satisfaction” and is commonly used to quantify the usability of systems. In addition to the usability of the system, the experienced task load can be measured by the National Aeronautics and Space Administration Task Load Index,^91^ which was used in three articles.

The use of AR technologies for surgical navigation and intraoperative guidance requires accurate and robust registration of the virtual overlay onto the real environment. Two commonly used distance measures for registration accuracy metrics are the FRE and the TRE. The FRE is a localization error between the location of a set of fiducial markers on a rigid base in physical space and in image space, defined as the total root mean square value of the errors measured per marker. The TRE is a measure for the registration accuracy onto a specific target point of interest (eg, a tumor). It is defined as the Euclidean distance between the physical target point of interest and the virtual point, which is measured in a 3D coordinate system. The FRE can be used to determine the accuracy of the fiducial registration and is useful in cases where the target point is attached to the same rigid base as the fiducial markers. In procedures where this is not the case and a high accuracy is essential, such as frameless stereotactic neurosurgery, the TRE is the recommended measure for image-to-patient registration.^96,97^ A frequently used subjective measure for registration accuracy was visual assessment. While relying solely on visual observation is not recommended, an advantage of using AR in surgery is the option for adjustment of the registration, for example, in the case of intraoperative brain shift. In addition, although few articles have reported this metric, the technical reliability of an AR system is important for its clinical applicability. This includes reporting on the system's stability through the on/off time ratio, the registration robustness through the successful registration ratio, and precision through the SD of the registration accuracy. The system's accuracy and reliability should be evaluated in a realistic OR setting before clinical implementation.

The evaluation of AR technologies for intraoperative guidance requires additional task-specific outcome metrics. Study designs should enable the measurement and potential comparison of task accuracy with and without AR guidance. Before clinical testing, in phase 2b (Figure 5), this is generally performed in phantoms. Phase 2b would be followed by the comparison of clinical outcomes in procedures with and without the use of AR guidance. This research has not reached the phases where cost-effectiveness and quality assurance regarding the technology and clinical benefits are measured yet. For future endeavors, Figure 5 provides an indication of the evaluation metrics that would be suitable for the following phases. For further clarification of Figure 5, an example of a phase 1 study researching surgical navigation would be the research by van Doormaal et al,^87^ who report on the FRE and the technical failures for their technology. An example of a phase 2a study researching preoperative visualization would be the research by Morales-Mojica et al,^26^ who report on system latencies and user perception. The studies by Van Gestel et al^19^ and Finger et al^70^ are interesting examples of a phase 2b/3 study researching intraoperative AR guidance, reporting on task accuracy measures and clinical performance.

Valorization and FDA approval of AR technology for surgical navigation and intraoperative guidance require the assessment of the safety and cost-effectiveness of AR technologies, demonstrating that the benefits in clinical practice outweigh the risks.^6^ For this purpose, we propose to report the outcomes of a study comparing AR use with non-AR use, eg, regarding surgical accuracy, procedure time, or clinical outcome. The evaluation metrics should accurately represent the risk profile and neurosurgical use case, eg, ventricular drain or craniotomy burr hole placement. Findings related to the stability and robustness of the AR system and any adverse health events encountered during its use should be reported. For compliance with Conformité Européenne certification and adherence to the European medical device regulation, the AR technology must satisfy the general safety and performance requirements specified in the medical device regulation, which also encompasses a clinical evaluation of the safety and performance of the system.^98^

### Study Limitations

This study has several limitations. The AR applications were categorized into three groups, and the evaluation metrics were grouped within these categories, based on frequency of reporting. Articles purely describing AR indications, without further mentioning evaluation measures, were not included in this review. If no explicit evaluation metrics regarding, eg, registration accuracy were mentioned but some distance measure was reported, it was classified under the general distance measure. The generalization allowed analysis of the evaluation metrics but might have resulted in some loss of specificity. The use of standardized evaluation metrics enables comparison of different approaches with a similar use case, but the balance between standardization and use case specificity should be taken into consideration for correct interpretation of Figure 5. In addition, the use of AR technologies in clinical practice might extend these three categories in the future, moving toward a “surgical cockpit” and, eg, incorporating performance feedback and intraoperative neuromonitoring. Furthermore, no quality of evidence assessment was applied in the present analysis. This is primarily attributed to a large heterogeneity in study designs and reporting practices, which presents challenges in conducting a standard quality of evidence assessment. Using a nonvalidated quality of evidence assessment could introduce bias, and thus also considering the aim of this review, no articles were excluded based on their study design and reporting style. However, in addition to Figure 5, we emphasize the importance for future research to prioritize unambiguous reporting and clear study designs because this would improve the overall quality of evidence in this field.

## CONCLUSION

This systematic review proposes an evaluation approach per neurosurgical AR application and innovation phase based on the current literature. To establish quality benchmarking and measure the value of AR technologies in neurosurgical practice, evaluation metrics should be specific to the technology's risk profile and clinical objectives. Key points include using validated questionnaires to assess user perception; ensuring clear and unambiguous reporting of registration accuracy, precision, robustness, and system stability; and accurately measuring task performance in clinical studies. Adopting these recommendations will enhance the reliability and validity of evaluations for AR technologies in neurosurgery, facilitating their integration into the clinical workflow and ultimately improving patient outcomes.

## Supplementary Material

SUPPLEMENTARY MATERIAL

## SUPPLEMENTAL DIGITAL CONTENT

**Supplemental digital content 1.** Search string. **Supplemental digital content 2, Table 1.** AR device used per study. **Supplemental digital content 3, Table 2.** Evaluation metrics per study. FRE = Fiducial Registration Error, TRE = Target Registration Error, TSR = Target Success Rate, VARA = Visual Assessment Registration Accuracy. **Supplemental digital content 4, Table 3.** AR applications for neurosurgical procedures.

## COMMENTS

Mixed reality (MxR), the union of virtual reality and augmented reality, is a technology that has come to stay as part of the neurosurgeon's armamentarium. These tools allow the surgeon to drive through the surgery “with GPS” and facilitate the correct development of the same.

This technology can also be used for morphometric studies, education and training, and patient engagement, among other applications. In my experience, MxR has changed my way of performing minimally invasive surgery, providing extra security in the procedure by being able to have a constant reference of the location of targets and antitargets or points to avoid. There are an increasing number of publications in the literature on its application in surgery, and especially in neurosurgery, but due to its novelty, there is a great heterogeneity in the nomenclature and parameters that are evaluated when performing these studies. This heterogeneity of terms leads to confusion and makes it impossible to develop solid evidence.

The authors present a manuscript of great value that attempts to homogenize these parameters. The inclusion of the IDEAL framework in the three surgical applications of MxR (preoperative visualization, surgical navigation, and intraoperative guidance) seems to be just that, ideal, for this purpose. In addition, the authors provide three examples to guide the reader who is interested in making a publication in relation to MxR, to choose the best possible performance parameters and nomenclature. This is an important task because the homogenization of these parameters will lead to more solid evidence in the application of this technology.

**Miguel Saez-Alegre**

*Rochester, Minnesota, USA*

## References

[1] Milgram P Kishino F. A taxonomy of mixed reality visual displays. IEICE Trans Inf Syst. 1994;77(12):1321-1329.

[2] Fiani B De Stefano F Kondilis A Covarrubias C Reier L Sarhadi K. Virtual reality in neurosurgery: “can you see it?”—a review of the current applications and future potential. World Neurosurg. 2020;141:291-298. 32561486 10.1016/j.wneu.2020.06.066

[3] Cannizzaro D Zaed I Safa A Augmented reality in neurosurgery, state of art and future projections. A systematic review. Front Surg. 2022;9:864792. 35360432 10.3389/fsurg.2022.864792 PMC8961734

[4] Durrani S Onyedimma C Jarrah R The virtual vision of neurosurgery: how augmented reality and virtual reality are transforming the neurosurgical operating room. World Neurosurg. 2022;168:190-201. 36208867 10.1016/j.wneu.2022.10.002

[5] Meola A Cutolo F Carbone M Cagnazzo F Ferrari M Ferrari V. Augmented reality in neurosurgery: a systematic review. Neurosurg Rev. 2017;40(4):537-548. 27154018 10.1007/s10143-016-0732-9 PMC6155988

[6] Administration USFD. Executive Summary for the Patient Engagement Advisory Committee Meeting—Augmented Reality and Virtual Reality Medical Devices. US Food & Drug Administration; 2022.

[7] Beams R Brown E Cheng W-C Evaluation challenges for the application of extended reality devices in medicine. J Digit Imaging. 2022;35(5):1409-1418. 35469355 10.1007/s10278-022-00622-x PMC9582055

[8] Page MA-O McKenzie JE Bossuyt PM The PRISMA 2020 statement: an updated guideline for reporting systematic reviews. BMJ. 2021;372. 10.1136/bmj.n71 PMC8005924 33782057

[9] Gsaxner C Li J Pepe A The HoloLens in medicine: a systematic review and taxonomy. Med Image Anal. 2023;85:102757. 36706637 10.1016/j.media.2023.102757

[10] Birkfellner W Figl M Matula C Computer-enhanced stereoscopic vision in a head-mounted operating binocular. Phys Med Biol. 2003;48(3):n49-n57. 12608617 10.1088/0031-9155/48/3/402

[11] Chiacchiaretta P Perrucci MG Caulo M A dedicated tool for presurgical mapping of brain tumors and mixed-reality navigation during neurosurgery. J Digit Imaging. 2022;35(3):704-713. 35230562 10.1007/s10278-022-00609-8 PMC9156583

[12] Coelho G Vieira EV Rabelo NN Preoperative planning modalities for meningoencephalocele: new proof of concept. World Neurosurg. 2021;151:124-131. 33964493 10.1016/j.wneu.2021.04.132

[13] Condino S Montemurro N Cattari N Evaluation of a wearable ar platform for guiding complex craniotomies in neurosurgery. Ann Biomed Eng. 2021;49(9):2590-2605. 34297263 10.1007/s10439-021-02834-8

[14] Creighton FX Unberath M Song T Zhao Z Armand M Carey J. Early feasibility studies of augmented reality navigation for lateral skull base surgery. Otol Neurotol. 2020;41(7):883-888. 32569148 10.1097/MAO.0000000000002724

[15] Cutolo F Meola A Carbone M A new head-mounted display-based augmented reality system in neurosurgical oncology: a study on phantom. Comput Assist Surg (Abingdon). 2017;22(1):39-53. 28754068 10.1080/24699322.2017.1358400

[16] Demerath T Stanicki A Roelz R Accuracy of augmented reality-guided drainage versus stereotactic and conventional puncture in an intracerebral hemorrhage phantom model. J Neurointerv Surg. 2023;15(7):708-711. 35853700 10.1136/neurintsurg-2022-018678 PMC10313981

[17] Fick T van Doormaal JAM Hoving EW Regli L van Doormaal TPC. Holographic patient tracking after bed movement for augmented reality neuronavigation using a head-mounted display. Acta Neurochir. 2021;163(4):879-884. 33515122 10.1007/s00701-021-04707-4 PMC7966201

[18] Van Gestel F Frantz T Soomro MH Augmented reality-assisted neurosurgical drain placement (ARANED): technical note. Acta Neurochir Suppl. 2021;131:267-273. 33839856 10.1007/978-3-030-59436-7_50

[19] Van Gestel F Frantz T Vannerom C The effect of augmented reality on the accuracy and learning curve of external ventricular drain placement. Neurosurg Focus. 2021;51(2):e8. 10.3171/2021.5.FOCUS21215 34333479

[20] Incekara F Smits M Dirven C Vincent A. Clinical feasibility of a wearable mixed-reality device in neurosurgery. World Neurosurg. 2018;118:e422-e427. 30257298 10.1016/j.wneu.2018.06.208

[21] Kubben P Sinlae R. Feasibility of using a low-cost head-mounted augmented reality device in the operating room. Surg Neurol Int. 2019;10(1):26. 31123633 10.4103/sni.sni_228_18 PMC6416754

[22] Li Y Chen X Wang N A wearable mixed-reality holographic computer for guiding external ventricular drain insertion at the bedside. J Neurosurg. 2019;131(5):1599-1606. 30485188 10.3171/2018.4.JNS18124

[23] Li Y Huang J Huang T Wearable mixed-reality holographic navigation guiding the management of penetrating intracranial injury caused by a nail. J Digit Imaging. 2021;34(2):362-366. 33846887 10.1007/s10278-021-00436-3 PMC8289971

[24] Maruyama K Watanabe E Kin T Smart glasses for neurosurgical navigation by augmented reality. Oper Neurosurg. 2018;15(5):551-556. 29373710 10.1093/ons/opx279

[25] Montemurro N Condino S Cattari N D'Amato R Ferrari V Cutolo F. Augmented reality-assisted craniotomy for parasagittal and convexity en plaque meningiomas and custom-made cranio-plasty: a preliminary laboratory report. Int J Environ Res Public Health. 2021;18(19):9955. 34639256 10.3390/ijerph18199955 PMC8507881

[26] Morales Mojica CM Velazco-Garcia JD Pappas EP A holographic augmented reality interface for visualizing of MRI data and planning of neurosurgical procedures. J Digit Imaging. 2021;34(4):1014-1025. 34027587 10.1007/s10278-020-00412-3 PMC8455790

[27] Neves CA Vaisbuch Y Leuze C Application of holographic augmented reality for external approaches to the frontal sinus. Int Forum Allergy Rhinol. 2020;10(7):920-925. 32362076 10.1002/alr.22546

[28] Peng C Yang L Yi W Application of fused reality holographic image and navigation technology in the puncture treatment of hypertensive intracerebral hemorrhage. Front Neurosci. 2022;16:850179. 35360174 10.3389/fnins.2022.850179 PMC8963409

[29] Qi Z Li Y Xu X Holographic mixed-reality neuronavigation with a head-mounted device: technical feasibility and clinical application. Neurosurg Focus. 2021;51(2):e22. 34333462 10.3171/2021.5.FOCUS21175

[30] Schneider M Kunz C Pal'a A Wirtz CR Mathis-Ullrich F Hlaváč M. Augmented reality-assisted ventriculostomy. Neurosurg Focus. 2021;50(1):e16. 10.3171/2020.10.FOCUS20779 33386016

[31] Stifano V Palumbo MC Chidambaram S The use of mixed reality for the treatment planning of unruptured intracranial aneurysms. J Neurosurg Sci. 2023;67(4):491-497. 34342192 10.23736/S0390-5616.21.05356-X

[32] Xu X Zheng Y Yao S Sun G Xu B Chen X. A low-cost multimodal head-mounted display system for neuroendoscopic surgery. Brain Behav. 2018;8(1):e00891. 29568688 10.1002/brb3.891 PMC5853619

[33] Yi Z Deng Z Liu Y Marker-less augmented reality based on monocular vision for falx meningioma localization. Int J Med Robot. 2022;18(1):e2341. 34647683 10.1002/rcs.2341

[34] Yoon JW Chen RE ReFaey K Technical feasibility and safety of image-guided parieto-occipital ventricular catheter placement with the assistance of a wearable head-up display. Int J Med Robot. 2017;13(4):e1836. 10.1002/rcs.1836 28524449

[35] Zhang ZY Duan WC Chen RK Preliminary application of mxed reality in neurosurgery: development and evaluation of a new intraoperative procedure. J Clin Neurosci. 2019;67:234-238. 31221576 10.1016/j.jocn.2019.05.038

[36] Zhou Z Yang Z Jiang S Zhuo J Zhu T Ma S. Augmented reality surgical navigation system based on the spatial drift compensation method for glioma resection surgery. Med Phys. 2022;49(6):3963-3979. 35383964 10.1002/mp.15650

[37] de Almeida AGC Fernandes de Oliveira Santos B Oliveira JLM. A neuronavigation system using a mobile augmented reality solution. World Neurosurg. 2022;167:e1261-e1267. 36089274 10.1016/j.wneu.2022.09.014

[38] Chen JG Han KW Zhang DF Li ZX Li YM Hou LJ. Presurgical planning for supratentorial lesions with Free Slicer Software and Sina App. World Neurosurg. 2017;106:193-197. 28673889 10.1016/j.wneu.2017.06.146

[39] Deng W Li F Wang M Song Z. Easy-to-use augmented reality neuronavigation using a wireless tablet PC. Stereotactic Funct Neurosurg. 2014;92(1):17-24. 10.1159/000354816 24216673

[40] Dho YS Park SJ Choi H Development of an inside-out augmented reality technique for neurosurgical navigation. Neurosurg Focus. 2021;51(2):E21. 34333463 10.3171/2021.5.FOCUS21184

[41] Eftekhar B. App-assisted external ventricular drain insertion. J Neurosurg. 2016;125(3):754-758. 26654178 10.3171/2015.6.JNS1588

[42] Eftekhar B. A Smartphone App to assist scalp localization of superficial supratentorial lesions-technical note. World Neurosurg. 2016;85:359-363. 26455767 10.1016/j.wneu.2015.09.091

[43] Hou Y Ma L Zhu R Chen X. iPhone-assisted augmented reality localization of basal ganglia hypertensive hematoma. World Neurosurg. 2016;94:480-492. 27449683 10.1016/j.wneu.2016.07.047

[44] Hou Y Ma L Zhu R Chen X Zhang J. A low-cost iPhone-assisted augmented reality solution for the localization of intracranial lesions. Plos One. 2016;11(7):e0159185. 27454518 10.1371/journal.pone.0159185 PMC4959690

[45] Léger É Reyes J Drouin S MARIN: an open-source mobile augmented reality interactive neuronavigation system. Int J Comput Assist Radiol Surg. 2020;15(6):1013-1021. 32323206 10.1007/s11548-020-02155-6

[46] Satoh M Nakajima T Yamaguchi T Watanabe E Kawai K. Evaluation of augmented-reality based navigation for brain tumor surgery. J Clin Neurosci. 2021;94:305-314. 34863455 10.1016/j.jocn.2021.10.033

[47] Shu XJ Wang Y Xin H Real-time augmented reality application in presurgical planning and lesion scalp localization by a smartphone. Acta Neurochir (Wien). 2022;164(4):1069-1078. 34448914 10.1007/s00701-021-04968-z

[48] Sun GC Chen XL Hou YZ Image-guided endoscopic surgery for spontaneous supratentorial intracerebral hematoma. J Neurosurg. 2017;127(3):537-542. 27636179 10.3171/2016.7.JNS16932

[49] Watanabe E Satoh M Konno T Hirai M Yamaguchi T. The trans-visible navigator: a see-through neuronavigation system using augmented reality. World Neurosurg. 2016;87:399-405. 26732958 10.1016/j.wneu.2015.11.084

[50] Asano K Katayama K Kakuta K Oyama K Ohkuma H. Assessment of the accuracy and errors of head-up display by an optical neuronavigation system in brain tumor surgery. Oper Neurosurg. 2017;13(1):23-35. 28931264 10.1093/ons/opw001

[51] Bárdosi Z Plattner C Özbek Y CIGuide: in situ augmented reality laser guidance. Int J Comput Assist Radiol Surg. 2020;15(1):49-57. 31506882 10.1007/s11548-019-02066-1 PMC6949325

[52] Bopp MHA Saß B Pojskić M Use of neuronavigation and augmented reality in transsphenoidal pituitary adenoma surgery. J Clin Med. 2022;11(19):5590. 36233457 10.3390/jcm11195590 PMC9571217

[53] Cabrilo I Bijlenga P Schaller K. Augmented reality in the surgery of cerebral aneurysms: a technical report. Neurosurgery. 2014;10(Suppl 2):252-260; discussion 260-1. 24594927 10.1227/NEU.0000000000000328

[54] Cabrilo I Bijlenga P Schaller K. Augmented reality in the surgery of cerebral arteriovenous malformations: technique assessment and considerations. Acta Neurochir. 2014;156(9):1769-1774. 25037466 10.1007/s00701-014-2183-9

[55] Cabrilo I Schaller K Bijlenga P. Augmented reality-assisted bypass surgery: embracing minimal invasiveness. World Neurosurg. 2015;83(4):596-602. 25527874 10.1016/j.wneu.2014.12.020

[56] Carl B Bopp M Voellger B Saß B Nimsky C. Augmented reality in transsphenoidal surgery. World Neurosurg. 2019;125:e873-e883. 30763743 10.1016/j.wneu.2019.01.202

[57] Caversaccio M Langlotz F Nolte LP Häusler R. Impact of a self-developed planning and self-constructed navigation system on skull base surgery: 10 years experience. Acta Otolaryngol. 2007;127(4):403-407. 17453461 10.1080/00016480601002104

[58] Davidovic A Chavaz L Meling TR Schaller K Bijlenga P Haemmerli J. Evaluation of the effect of standard neuronavigation and augmented reality on the integrity of the perifocal structures during a neurosurgical approach. Neurosurg Focus. 2021;51(2):e19. 34333474 10.3171/2021.5.FOCUS21202

[59] Eljamel MS. Frameless stereotactic neurosurgery: two steps towards the Holy Grail of surgical navigation. Stereotact Funct Neurosurg. 1999;72(2-4):125-128. 10.1159/000029711 10853063

[60] Haemmerli J Davidovic A Meling TR Chavaz L Schaller K Bijlenga P. Evaluation of the precision of operative augmented reality compared to standard neuronavigation using a 3D-printed skull. Neurosurg Focus. 2021;50(1):e17. 10.3171/2020.10.FOCUS20789 33386018

[61] King AP Edwards PJ Maurer CR Jr. A system for microscope-assisted guided interventions. Stereotact Funct Neurosurg. 1999;72(2-4):107-111. 10853060 10.1159/000029708

[62] Louis RG Steinberg GK Duma C Early experience with virtual and synchronized augmented reality platform for preoperative planning and intraoperative navigation: a case series. Oper Neurosurg. 2021;21(4):189-196. 34171909 10.1093/ons/opab188 PMC8453400

[63] Mascitelli JR Schlachter L Chartrain AG Navigation-linked heads-up display in intracranial surgery: early experience. Oper Neurosurg. 2018;15(2):184-193. 29040677 10.1093/ons/opx205 PMC6047456

[64] Paul P Fleig O Jannin P. Augmented virtuality based on stereoscopic reconstruction in multimodal image-guided neurosurgery: methods and performance evaluation. IEEE Trans Med Imaging. 2005;24(11):1500-1511. 16279086 10.1109/TMI.2005.857029

[65] Pojskić M Bopp MHA Saβ B Carl B Nimsky C. Microscope-based augmented reality with intraoperative computed tomography-based navigation for resection of skull base meningiomas in consecutive series of 39 patients. Cancers. 2022;14(9):2302. 35565431 10.3390/cancers14092302 PMC9101634

[66] Roethe AL Rösler J Misch M Vajkoczy P Picht T. Augmented reality visualization in brain lesions: a prospective randomized controlled evaluation of its potential and current limitations in navigated microneurosurgery. Acta Neurochir. 2022;164(1):3-14. 34904183 10.1007/s00701-021-05045-1 PMC8761141

[67] Sun GC Wang F Chen XL Impact of virtual and augmented reality based on intraoperative magnetic resonance imaging and functional neuronavigation in glioma surgery involving eloquent areas. World Neurosurg. 2016;96:375-382. 27521727 10.1016/j.wneu.2016.07.107

[68] Toyooka T Otani N Wada K Head-up display may facilitate safe keyhole surgery for cerebral aneurysm clipping. J Neurosurg. 2018;129(4):883-889. 29192858 10.3171/2017.5.JNS162692

[69] Dixon BJ Daly MJ Chan H Vescan A Witterick IJ Irish JC. Augmented image guidance improves skull base navigation and reduces task workload in trainees: a preclinical trial. Laryngoscope. 2011;121(10):2060-2064. 21898439 10.1002/lary.22153

[70] Finger T Schaumann A Schulz M Thomale UW. Augmented reality in intraventricular neuroendoscopy. Acta Neurochir. 2017;159(6):1033-1041. 28389876 10.1007/s00701-017-3152-x

[71] Lai M Skyrman S Shan C Fusion of augmented reality imaging with the endoscopic view for endonasal skull base surgery; a novel application for surgical navigation based on intraoperative cone beam computed tomography and optical tracking. PLoS One. 2020;15(1):e0227312. 31945082 10.1371/journal.pone.0227312 PMC6964902

[72] Li L Yang J Chu Y A novel augmented reality navigation system for endoscopic sinus and skull base surgery: a feasibility study. PLoS One. 2016;11(1):e0146996. 26757365 10.1371/journal.pone.0146996 PMC4710572

[73] Marcus HJ Pratt P Hughes-Hallett A Comparative effectiveness and safety of image guidance systems in surgery: a preclinical randomised study. Lancet. 2015;385(Suppl 1):s64. 26312886 10.1016/S0140-6736(15)60379-8

[74] Zeiger J Costa A Bederson J Shrivastava RK Iloreta AMC. Use of mixed reality visualization in endoscopic endonasal skull base surgery. *Oper Neurosurg*. 2020;19(1):43-52. 31807786 10.1093/ons/opz355

[75] Zhu T Jiang S Yang Z A neuroendoscopic navigation system based on dual-mode augmented reality for minimally invasive surgical treatment of hypertensive intracerebral hemorrhage. Comput Biol Med. 2022;140:105091. 34872012 10.1016/j.compbiomed.2021.105091

[76] Gerard IJ Kersten-Oertel M Drouin S Combining intraoperative ultrasound brain shift correction and augmented reality visualizations: a pilot study of eight cases. J Med Imaging. 2018;5(2):021210. 10.1117/1.JMI.5.2.021210 PMC5786643 29392162

[77] Kersten-Oertel M Chen SS Drouin S Sinclair DS Collins DL. Augmented reality visualization for guidance in neurovascular surgery. Stud Health Technol Inform. 2012;173:225-229. 22356991

[78] Kersten-Oertel M Gerard I Drouin S Augmented reality in neurovascular surgery: feasibility and first uses in the operating room. Int J Comput Assist Radiol Surg. 2015;10(11):1823-1836. 25712917 10.1007/s11548-015-1163-8

[79] Kockro RA Tsai YT Ng I DEX-Ray: augmented reality neurosurgical navigation with a handheld video probe. Neurosurgery. 2009;65(4):795-807; discussion 807-8. 19834386 10.1227/01.NEU.0000349918.36700.1C

[80] Low D Lee CK Dip LL Ng WH Ang BT Ng I. Augmented reality neurosurgical planning and navigation for surgical excision of parasagittal, falcine and convexity meningiomas. Br J Neurosurg. 2010;24(1):69-74. 20158356 10.3109/02688690903506093

[81] Pandya A Siadat MR Auner G Kalash M Ellis RD. Development and human factors analysis of neuronavigation vs. augmented reality. Stud Health Technol Inform. 2004;98:291-297. 15544292

[82] Skyrman S Lai M Edström E Augmented reality navigation for cranial biopsy and external ventricular drain insertion. Neurosurg Focus. 2021;51(2):e7. 10.3171/2021.5.FOCUS20813 34333469

[83] Yavas G Caliskan KE Cagli MS. Three-dimensional-printed marker-based augmented reality neuronavigation: a new neuronavigation technique. Neurosurg Focus. 2021;51(2):e20. 34333464 10.3171/2021.5.FOCUS21206

[84] Besharati Tabrizi L Mahvash M. Augmented reality-guided neurosurgery: accuracy and intraoperative application of an image projection technique. J Neurosurg. 2015;123(1):206-211. 25748303 10.3171/2014.9.JNS141001

[85] Wu B Liu P Xiong C Stereotactic co-axial projection imaging for augmented reality neuronavigation: a proof-of-concept study. Quant Imaging Med Surg. 2022;12(7):3792-3802. 35782260 10.21037/qims-21-1144 PMC9246757

[86] Zeng B Meng F Ding H Wang G. A surgical robot with augmented reality visualization for stereoelectroencephalography electrode implantation. Int J Comput Assist Radiol Surg. 2017;12(8):1355-1368. 28664416 10.1007/s11548-017-1634-1

[87] van Doormaal TPC van Doormaal JAM Mensink T. Clinical accuracy of holographic navigation using point-based registration on augmented-reality glasses. Oper Neurosurg. 2019;17(6):588-593. 31081883 10.1093/ons/opz094 PMC6995446

[88] Gibby W Cvetko S Gibby A The application of augmented reality-based navigation for accurate target acquisition of deep brain sites: advances in neurosurgical guidance. J Neurosurg. 2022;137(2):489-495. 34920422 10.3171/2021.9.JNS21510

[89] Ivan ME Eichberg DG Di L Augmented reality head-mounted display-based incision planning in cranial neurosurgery: a prospective pilot study. Neurosurg Focus. 2021;51(2):e3. 10.3171/2021.5.FOCUS20735 34333466

[90] Besharati Tabrizi L Mahvash M. Augmented reality-guided neurosurgery: accuracy and intraoperative application of an image projection technique. J Neurosurg. 2015;123(1):206-211. 25748303 10.3171/2014.9.JNS141001

[91] Hart SG Staveland LE. Development of NASA-TLX (Task Load Index): results of empirical and theoretical research. Human mental workload. In: Advances in Psychology, Vol 52. North-Holland; 1988:139-183.

[92] Mincu D Roy S. Developing robust benchmarks for driving forward AI innovation in healthcare. Nat Mach Intell. 2022;4(11):916-921.

[93] Hicks SA Strümke I Thambawita V On evaluation metrics for medical applications of artificial intelligence. Sci Rep. 2022;12(1):5979. 35395867 10.1038/s41598-022-09954-8 PMC8993826

[94] McCulloch P Altman DG Campbell WB No surgical innovation without evaluation: the IDEAL recommendations. Lancet. 2009;374(9695):1105-1112. 19782876 10.1016/S0140-6736(09)61116-8

[95] Lund A. Measuring Usability with the USE Questionnaire. STC Usability SIG Newsletter. 8(2):3-6.

[96] Fitzpatrick JM. Fiducial registration error and target registration error are uncorrelated. Proceedings Volume 7261, Medical Imaging 2009: Visualization, Image-Guided Procedures, and Modeling. 2009;726102.

[97] Woerdeman PA Willems PWA Noordmans HJ Tulleken CAF van der Sprenkel JWB. Application accuracy in frameless image-guided neurosurgery: a comparison study of three patient-to-image registration methods. J Neurosurg. 2007;106(6):1012-1016. 17564173 10.3171/jns.2007.106.6.1012

[98] The European Union Medical Device Regulation—MDCG 2021-8 Annex 6. 2021. Accessed June 15, 2023.

