# Supplementary material for: Evaluation Metrics for Augmented Reality in Neurosurgical Preoperative Planning, Surgical Navigation, and Surgical Treatment Guidance: A Systematic Review
Source: Oper Neurosurg. 2023 Dec 26;26(5):491–501. doi: 10.1227/ons.0000000000001009 (PMC11008635; doi:10.1227/ons.0000000000001009)
Supplement: SUPPLEMENTARY MATERIAL [file ons-26-491-s001.docx]

### Supplemental Digital Content 1 – Search String

**Pubmed (363)**
("neurosurg*"[Title/Abstract] OR "Neurosurgery"[MeSH Terms] OR "Neurosurgical Procedures"[MeSH Terms] OR "ventriculostom*"[Title/Abstract] OR "lobectom*"[Title/Abstract] OR "craniotom*"[Title/Abstract] OR "neuro surg*"[Title/Abstract] OR "neurologic surg*"[Title/Abstract]) AND ("augmented realit*"[Title/Abstract] OR "Augmented Reality"[MeSH Terms] OR "mixed realit*"[Title/Abstract] OR "extended realit*"[Title/Abstract] OR "haptic technolog*"[Title/Abstract] OR "hologra*"[Title/Abstract] OR "Holography"[MeSH Terms] OR "head mounted display*"[Title/Abstract] OR "head up display*"[Title/Abstract] OR "Smart Glasses"[MeSH Terms])

**Embase (460)**('neurosurg*':ti,ab,kw OR 'neurosurgery'/exp OR 'ventriculostom*':ti,ab,kw OR 'lobectom*':ti,ab,kw OR 'craniotom*':ti,ab,kw OR 'neuro surg*':ti,ab,kw OR 'neurologic surg*':ti,ab,kw) AND ('augmented realit*':ti,ab,kw OR 'augmented reality'/exp OR 'mixed realit*':ti,ab,kw OR 'extended realit*':ti,ab,kw OR 'haptic technolog*':ti,ab,kw OR 'hologra*':ti,ab,kw OR 'holography'/exp OR 'head mounted display*':ti,ab,kw OR 'head up display*':ti,ab,kw OR 'smart glasses'/exp OR 'augmented reality system'/exp)

**Cochrane (7)**((neurosurg* NEXT procedur*):ti,ab,kw OR (neurosurger*):ti,ab,kw OR (neurosurgery)/exp OR (ventriculostom*):ti,ab,kw OR (lobectom*):ti,ab,kw OR (craniotom*):ti,ab,kw OR (neuro NEXT surg*):ti,ab,kw OR (neurologic NEXT surg):ti,ab,kw)) AND ((augmented reality)/exp OR (augmented NEXT realit*):ti,ab,kw OR (mixed NEXT realit*):ti,ab,kw OR (extended NEXT realit*):ti,ab,kw OR (haptic NEXT technolog*):ti,ab,kw OR (head up display*):ti,ab,kw OR (smart glas*)/exp OR (augmented reality NEXT system*)/exp
