# Supplementary material for: Evaluation Metrics for Augmented Reality in Neurosurgical Preoperative Planning, Surgical Navigation, and Surgical Treatment Guidance: A Systematic Review
Source: Oper Neurosurg. 2023 Dec 26;26(5):491–501. doi: 10.1227/ons.0000000000001009 (PMC11008635; doi:10.1227/ons.0000000000001009)
Supplement: SUPPLEMENTARY MATERIAL [file ons-26-491-s002.docx]

### Supplemental Digital Content 2 – Table 1

**Table 1.** AR device used per study.

| Device | | Studies |
| --- | --- | --- |
| *Head-mounted device (30)* | | Birkfellner et al. ^10^, Chiacchiaretta et al. ^11^, Coelho et al. ^12^, Condino et al. ^13^, Creighton et al. ^14^, Cutolo et al. ^15^, Demerath et al. ^16^, van Doormaal et al. ^17^, van Gestel et al. ^18^, van Gestel et al. ^19^, Gibby et al. ^20^, Ivan et al. ^21^, Li et al. ^22^, Li et al. ^23^, Maruyama et al. ^24^, Montemurro et al. ^25^, Morales Mojica et al. ^26^, Neves et al. ^27^, Peng et al. ^28^, Qi et al. ^29^, Schneider et al. ^30^, Stifano et al.e ^31^, Xu et al. ^32^, Yi et al. ^33^, Yoon et al. ^34^, Zhang et al. ^35^, Zhou et al. ^36^ |
| *Handheld device (13)* | | de Almeida et al. ^37^, Chen et al. ^38^, Deng et al. ^39^, Dho et al. ^40^, Eftekhar et al. ^41^, Eftekhar et al. ^42^, Hou et al. ^43^, Hou et al. ^44^, Léger et al. ^45^, Satoh et al. ^46^, Shu et al. ^47^, Sun et al. ^48^, Watanabe et al. ^49^ |
| *Image overlay (26)* | *Microscope (19)* | Asano et al. ^50^, Bardosi et al. ^51^, Bopp et al. ^52^, Cabrilo et al. ^53^, Cabrilo et al. ^54^, Cabrilo et al. ^55^, Carl et al. ^56^, Caversaccio et al. ^57^, Davidovic et al. ^58^, Eljamel et al. ^59^, Haemmerli et al. ^60^, King et al. ^61^, Louis et al. ^62^, Mascitelli et al. ^63^, Paul et al. ^64^, Pojskić et al. ^65^, Roethe et al. ^66^, Sun et al. ^67^, Toyooka et al. ^68^ |
|  | *Neuro- endoscope (7)* | Dixon et al ^69^, Finger et al. ^70^, Lai et al. ^71^, Li et al. ^72^, Marcus et al. ^73^, Zeiger et al. ^74^, Zhu et al. ^75^ |
| *External camera/*  *projector equipment (11)* | *Camera (8)* | Gerard et al. ^76^, Kersten-Oertel et al. ^77^, Kersten-Oertel et al. ^78^, Kockro et al. ^79^, Low et al. ^80^, Pandya et al. ^81^, Skyrman et al. ^82^, Yavas et al. ^83^ |
|  | *Projector (3)* | Tabrizi et al. ^84^, Wu et al. ^85^, Zeng et al. ^86^ |
