# Supplementary material for: Evaluation Metrics for Augmented Reality in Neurosurgical Preoperative Planning, Surgical Navigation, and Surgical Treatment Guidance: A Systematic Review
Source: Oper Neurosurg. 2023 Dec 26;26(5):491–501. doi: 10.1227/ons.0000000000001009 (PMC11008635; doi:10.1227/ons.0000000000001009)
Supplement: SUPPLEMENTARY MATERIAL [file ons-26-491-s003.docx]

### Supplemental Digital Content 3 – Table 2

**Table 2.** Evaluation metrics per study. FRE = Fiducial Registration Error, TRE = Target Registration Error, TSR = Target Success Rate, VARA = Visual Assessment Registration Accuracy.

| Study | Evaluation metrics | |
| --- | --- | --- |
| De Almeida et al. ^37^ | *Quantitative* | TRE |
| Asano et al. ^50^ | *Quantitative* | Tumor removal  Preservation important structures |
|  | *Qualitative* | VARA |
| Bárdosi et al. ^51^ | *Quantitative* | TRE |
| Birkfellner et al. ^10^ | *Quantitative* | FRE  Task success rate |
| Bopp et al. ^52^ | *Quantitative* | TRE  Registration time  Planning time  Operative time |
|  | *Qualitative* | Complication occurrence  AR indications |
| Cabrilo et al. ^53^ | *Qualitative* | VARA  Usefulness evaluation |
| Cabrilo et al. ^54^ | *Qualitative* | VARA  Usefulness evaluation |
| Cabrilo et al. ^55^ | *Qualitative* | VARA  Usefulness evaluation |
| Carl et al. ^56^ | *Quantitative* | TRE |
|  | *Qualitative* | Usefulness evaluation |
| Caversaccio et al. ^57^ | *Quantitative* | FRE  Operative time |
|  | *Qualitative* | Complication occurrence |
| Chen et al. ^38^ | *Quantitative* | Distance measure  Registration time |
| Chiacchiaretta et al. ^11^ | *Qualitative* | VARA  Usability evaluation |
| Coelho et al. ^12^ | *Qualitative* | Complication occurrence |
| Condino et al. ^13^ | *Quantitative* | TRE  Task accuracy |
|  | *Qualitative* | Clinical feasibility |
| Creighton et al. ^14^ | *Quantitative* | TRE |
| Cutolo et al. ^15^ | *Quantitative* | Task success rate |
|  | *Qualitative* | Ergonomics evaluation  Depth perception |
| Davidovic et al. ^58^ | *Quantitative* | Task success rate  Craniotomy size |
|  | *Qualitative* | Trust in system |
| Demerath et al. ^16^ | *Quantitative* | TRE |
| Deng et al. ^39^ | *Quantitative* | Distance measure |
|  | *Qualitative* | Clinical feasibility |
| Dho et al. ^40^ | *Quantitative* | Distance measure |
| Dixon et al. ^69^ | *Quantitative* | FRE |
|  | *Qualitative* | Task load questionnaire (NASA-TLX) |
| Van Doormaal et al. ^87^ | *Quantitative* | FRE  Technical failures |
| Eftekhar et al. ^41^ | *Quantitative* | Distance measure  Target point deviation |
|  | *Qualitative* | Usability evaluation |
| Eftekhar et al. ^42^ | *Quantitative* | Distance measure  Registration time |
| Eljamel et al. ^59^ | *Quantitative* | Distance measure |
| Fick et al. ^17^ | *Quantitative* | FRE |
| Finger et al. ^70^ | *Quantitative* | Distance measure  Target point deviation  Registration time |
|  | *Qualitative* | Performance evaluation  Clinical outcome  Complication occurrence |
| Gerard et al. ^76^ | *Qualitative* | Usability evaluation |
| Van Gestel et al. ^18^ | *Quantitative* | Planning time |
|  | *Qualitative* | Usability evaluation (comments) |
| Van Gestel et al. ^19^ | *Quantitative* | Target point deviation  Task success rate  Spatial aptitude |
|  | *Qualitative* | Usability evaluation  Qualitative task performance |
| Gibby et al. ^88^ | *Quantitative* | Distance measure  Target angle deviation  Target point deviation |
| Haemmerli et al. ^60^ | *Quantitative* | Target point deviation |
|  | *Qualitative* | Trust in system |
| Hou et al. ^43^ | *Quantitative* | Distance measure  Target angle deviation  Target point deviation |
| Hou et al. ^44^ | *Quantitative* | Target angle deviation  Distance measure |
| Incekara et al. ^20^ | *Quantitative* | Distance measure  Planning time |
|  | *Qualitative* | Ergonomics |
| Ivan et al. ^89^ | *Quantitative* | Overlap percentage |
| Kersten-Oertel et al. ^77^ | *Quantitative* | FRE  Distance measure  VARA |
|  | *Qualitative* | Usefulness evaluation  Depth perception |
| Kersten-Oertel et al. ^78^ | *Qualitative* | Usability evaluation (comments) |
| King et al. ^61^ | *Quantitative* | TRE  Distance measure |
| Kockro et al. ^79^ | *Quantitative* | FRE  Registration time |
|  | *Qualitative* | Performance evaluation |
| Kubben et al. ^21^ | *Qualitative* | Performance evaluation |
| Lai et al. ^71^ | *Quantitative* | TRE |
| Leger et al. ^45^ | *Quantitative* | Time for task  Target point deviation |
|  | *Qualitative* | Task load questionnaire (NASA-TLX) |
| Li et al. ^72^ | *Quantitative* | TRE |
|  | *Qualitative* | Task load questionnaire (NASA-TLX) |
| Li et al. ^22^ | *Quantitative* | Task success rate  Target point deviation  Time for task  Technical failures |
|  | *Qualitative* | Complication occurrence |
| Li et al. ^23^ | *Qualitative* | Clinical outcome case report |
| Louis at al. ^62^ | *Quantitative* | Planning time |
|  | *Qualitative* | VARA  Usefulness evaluation |
| Low et al. ^80^ | *Qualitative* | Usefulness evaluation  Correspondence with intraoperative findings |
| Marcus et al. ^73^ | *Quantitative* | Target point deviation  Time for task |
|  | *Qualitative* | Depth perception  Recognition of unexpected finding |
| Maruyama et al. ^24^ | *Quantitative* | TRE  Registration time |
|  | *Qualitative* | Tumor removal rate  Complication occurrence |
| Mascitelli et al. ^63^ | *Qualitative* | VARA  Technical failures  Usability evaluation |
| Montemurro et al. ^25^ | *Quantitative* | Distance measure  Task accuracy |
|  | *Qualitative* | Ergonomics evaluation |
| Morales Mojica et al. ^26^ | *Quantitative* | System latency |
|  | *Qualitative* | Ergonomics evaluation  Usability evaluation |
| Neves et al. ^27^ | *Quantitative* | Distance measure  Registration time |
|  | *Qualitative* | Complication occurrence |
| Pandya et al. ^81^ | *Quantitative* | Distance measure  Spatial aptitude  Craniotomy size |
|  | *Qualitative* | Usefulness evaluation |
| Paul et al. ^64^ | *Quantitative* | Distance measure  Robustness |
| Peng et al. ^28^ | *Quantitative* | Task success rate  Operative time |
|  | *Qualitative* | Complication occurrence |
| Pojskić et al. ^65^ | *Quantitative* | TRE  Operative time |
|  | *Qualitative* | Usability evaluation  AR indications  Volume measurements  Tumor removal  Complication occurrence |
| Qi et al. ^29^ | *Quantitative* | FRE  Comparison NN  Registration time |
| Roethe et al. ^66^ | *Qualitative* | VARA  Performance evaluation  Spatial aptitude |
| Satoh et al. ^46^ | *Quantitative* | Comparison NN |
|  | *Qualitative* | Usefulness evaluation |
| Schneider et al. ^30^ | *Quantitative* | Target point deviation  Task success rate |
|  | *Qualitative* | Usability evaluation |
| Shu et al. ^47^ | *Quantitative* | FRE  Registration time  Distance measure  Comparison NN  Area Overlap Rate |
| Skyrman et al. ^82^ | *Quantitative* | Target point deviation  Target angle deviation  Time for task  Task success rate |
| Stifano et al. ^31^ | *Qualitative* | Usability evaluation  Ergonomics evaluation  Performance evaluation |
| Sun et al. ^67^ | *Quantitative* | Volume measurements |
|  | *Qualitative* | Surgical approach  Tumor removal  Clinical outcome |
| Sun et al. ^48^ | *Quantitative* | Operative time  Volume measurements |
|  | *Qualitative* | Clinical outcome |
| Tabrizi et al. ^90^ | *Quantitative* | Distance measure  Registration time |
|  | *Qualitative* | Clinical reliability  Tumor removal |
| Toyooka et al. ^68^ | *Quantitative* | Craniotomy size  Operative time |
|  | *Qualitative* | Clinical outcome |
| Watanabe et al. ^49^ | *Quantitative* | Distance measure  Registration time |
|  | *Qualitative* | Clinical feasibility |
| Wu et al. ^85^ | *Quantitative* | Distance measure  Registration time  Operative time  Planning time |
| Xu et al. ^32^ | *Qualitative* | System-related complications  Clinical feasibility  Ergonomics evaluation  Clinical outcome (ventricular diseases)  Complication occurrence |
| Yavas et al. ^83^ | *Quantitative* | Distance measure  Planning time |
|  | *Qualitative* | Tumor removal  Complication occurrence |
| Yi et al. ^33^ | *Quantitative* | TRE  Planning time  Precision |
| Yoon et al.^34^ | *Quantitative* | Task success rate |
|  | *Qualitative* | Complication occurrence |
| Zeiger et al. ^74^ | *Quantitative* | Operative time  Performance (number of times AR abandonment) |
|  | *Qualitative* | Technical failures  Performance evaluation  Complication occurrence  Tumor removal |
| Zeng et al. ^86^ | *Quantitative* | FRE  TRE |
|  | *Qualitative* | VARA |
| Zhang et al. ^35^ | *Qualitative* | Clinical feasibility |
| Zhou et al. ^36^ | *Quantitative* | FRE  Registration time |
| Zhu et al. ^75^ | *Quantitative* | Registration accuracy (FRE, pixel match ratio error %)  Precision  Target angle deviation  Target point deviation  Operative time  Tumor removal |
