# Supplementary material for: Evaluation Metrics for Augmented Reality in Neurosurgical Preoperative Planning, Surgical Navigation, and Surgical Treatment Guidance: A Systematic Review
Source: Oper Neurosurg. 2023 Dec 26;26(5):491–501. doi: 10.1227/ons.0000000000001009 (PMC11008635; doi:10.1227/ons.0000000000001009)
Supplement: SUPPLEMENTARY MATERIAL [file ons-26-491-s004.docx]

### Supplemental Digital Content 4 – Table 3

***Table 3****.* AR applications for neurosurgical procedures.

| Application | Studies |
| --- | --- |
| Preoperative visualization (4) | Coelho et al. ^12^, Kubben et al. ^21^, Morales Mojica et al. ^26^, Zhang et al. ^35^ |
| Surgical navigation (60) | Asano et al. ^50^, Bárdosi et al. ^51^, Bopp et al. ^52^, Cabrilo et al. ^53^, Cabrilo et al. ^54^, Cabrilo et al. ^55^, Carl et al. ^56^, Caversaccio et al. ^57^, Chen et al. ^38^, Chiacchiaretta et al. ^11^, Condino et al. ^13^, Davidovic et al. ^58^, Demerath et al. ^16^, Deng et al. ^39^, Dho et al. ^40^, Dixon et al. ^69^, van Doormaal et al. ^87^, Eftekhar et al. ^41^, Eftekhar et al. ^42^, Eljamel et al. ^59^, Fick et al. ^17^, Gibby et al. ^88^, Haemmerli et al. ^60^, Hou et al. ^43^, Hou et al. ^44^, Incekara et al. ^20^, Ivan et al. ^89^, King et al. ^61^, Lai et al, ^71^, Li et al. ^72^, Li et al. ^22^, Li et al. ^23^, Louis et al. ^62^, Maruyama et al. ^24^, Mascitelli et al. ^63^, Montemurro et al. ^25^, Neves et al. ^27^, Paul et al. ^64^, Peng et al. ^28^, Pojskić et al. ^65^, Qi et al. ^29^, Roethe et al. ^66^, Satoh et al. ^46^, Schneider et al. ^30^, Shu et al. ^47^, Skyrman et al. ^82^, Stifano et al. ^31^, Sun et al. ^67^, Sun et al. ^48^, Tabrizi et al. ^90^, Toyooka et al. ^68^, Watanabe et al. ^49^, Wu et al. ^85^, Xu et al. ^32^, Yavas et al. ^83^, Yi et al. ^33^, Zeiger et al. ^74^, Zeng et al. ^86^, Zhou et al. ^36^, Zhu et al. ^75^ |
| Intraoperative guidance (16) | de Almeida et al. ^37^, Birkfellner et al. ^10^, Creighton et al. ^14^, Cutolo et al. ^15^, Finger et al. ^70^, Gerard et al. ^76^, van Gestel et al. ^19^, van Gestel et al. ^18^, Kersten-Oertel et al. ^77^, Kersten-Oertel et al. ^78^, Kockro et al. ^79^, Léger et al. ^45^, Low et al. ^80^, Marcus et al. ^73^, Pandya et al. ^81^, Yoon et al. ^34^ |
